# Methods for the evaluation of hospital cooperation activities (Systematic review protocol)

**DOI:** 10.1186/2046-4053-1-11

**Published:** 2012-02-10

**Authors:** Thomas Rotter, Daniela Popa, Beatrice Riley, Tim Ellermann, Ulrike Ryll, Genc Burazeri, Piet Daemen, Guy Peeters, Helmut Brand

**Affiliations:** 1 Department of International Health, School for Public Health and Primary Care (CAPHRI), Faculty of Health, Medicine & Life Sciences, Maastricht University, Maastricht, The Netherlands; 2 Maastricht University Medical Centre + (UMC+), Maastricht, The Netherlands

## Abstract

**Background:**

Hospital partnerships, mergers and cooperatives are arrangements frequently seen as a means of improving health service delivery. Many of the assumptions used in planning hospital cooperatives are not stated clearly and are often based on limited or poor scientific evidence.

**Methods:**

This is a protocol for a systematic review, following the Cochrane EPOC methodology. The review aims to document, catalogue and synthesize the existing literature on the reported methods for the evaluation of hospital cooperation activities as well as methods of hospital cooperation. We will search the Database of Abstracts of Reviews of Effectiveness, the Effective Practice and Organisation of Care Register, the Cochrane Central Register of Controlled Trials and bibliographic databases including PubMed (via NLM), Web of Science, NHS EED, Business Source Premier (via EBSCO) and Global Health for publications that report on methods for evaluating hospital cooperatives, strategic partnerships, mergers, alliances, networks and related activities and methods used for such partnerships. The method proposed by the Cochrane EPOC group regarding randomized study designs, controlled clinical trials, controlled before and after studies, and interrupted time series will be followed. In addition, we will also include cohort, case-control studies, and relevant non-comparative publications such as case reports. We will categorize and analyze the review findings according to the study design employed, the study quality (low versus high quality studies) and the method reported in the primary studies. We will present the results of studies in tabular form.

**Discussion:**

Overall, the systematic review aims to identify, assess and synthesize the evidence to underpin hospital cooperation activities as defined in this protocol. As a result, the review will provide an evidence base for partnerships, alliances or other fields of cooperation in a hospital setting. PROSPERO registration number: CRD42011001579

## Background

Research into the ways hospital services are managed, into understanding hospital cooperatives, hospital partnerships, or for instance hospital mergers has, for long, been neglected [1-3]. This is astonishing since hospitals account for 40% to 60% of the health expenditure in OECD countries [4]. From a management perspective, the structure and organization of hospitals and related fields of health care has increasingly experienced a shift from independent ownerships to inter-organizational relationships. Those hospital cooperation/partnership activities are the result of an attempt to optimize structures and processes in terms of the public health and hospital stakeholders to increase the effectiveness of the hospital system. Many of the assumptions used in planning hospital cooperatives are not stated clearly and are often based on limited or poor scientific evidence [3].

Therefore, it is time to document, catalogue and synthesize the existing literature via a systematic review on the reported methods for the evaluation of hospital cooperation activities as well as objectively reported methods of hospital cooperation. We will exclude subjective methods or measures reported such as expert opinion or self reported satisfaction. The systematic review should also provide an evidence base for the already existing cooperation between the university hospitals in Aachen, Germany, and Maastricht, The Netherlands. Both hospitals intend to intensify their cooperation, aiming towards the founding of a European cross-border University Hospital Aachen-Maastricht [5].

The concept of hospital cooperation relates to all information that is included in the terms 'Hospital Cooperation', 'Health Facility Merger', 'Hospital Shared Services', 'Health Care Coalitions' and 'Health Facility Moving'[6]. Thus, the review question shall be broadly inclusive so that all possible literature will be screened for inclusion. For the purpose of this review, the concept of hospital cooperation includes the following definitions:

• Hospital-Cooperation

Includes the concepts hospital and cooperation [6]. Hospitals are institutions which provide medical care to patients [6]. The concept cooperation refers to international cooperation activities as well as cooperative behavior, multi-institutional systems, and organizational affiliation [6]. These activities also include alliances, partnerships and networks in various ways, based on the aims of the collaboration (see also additional file 1, search concepts for PubMed).

• Health Facility and Hospital Merger

The combining of administrative and organizational resources of two or more health care facilities [6].

• Hospital Shared Service

Cooperation among hospitals for the purpose of sharing various departmental services, for example, pharmacy, laundry, data processing, and so on [6].

• Health Care Coalitions

Voluntary groups of people representing diverse interests in the community such as hospitals, businesses, physicians, and insurers [6].

• Health Facility Moving

The relocation of health care institutions or units thereof. The concept includes equipment relocation [6].

• Fusion

Fusion is defined as a merging of diverse elements into a unified whole [7]. For the purpose of this review, we refer to hospital fusion as a process of fusion of two or more hospital facilities.

### Review questions and objectives

The review questions are as follows:

(i) What are the reported methods for evaluating hospital cooperation activities?

(ii) What are the reported methods of hospital cooperation including different levels of infrastructure?

(iii) What are the reported effects of hospital cooperation on professional practice and patient outcomes, including economic measures (that is, quality of care, hospital costs, charges, and so on)?

Overall, the systematic review aims to identify, assess and synthesize evidence to underpin activities of hospital partnerships, cooperatives, and mergers.

### Criteria for considering existing publications for this review

As the systematic review will include all relevant publications according to the review question and objectives, we expect a diverse range of different settings and evaluations contributing information on possible hospital cooperation methods, strategies, and the possible outcomes.

### Types of publications/studies

The method proposed by the Cochrane Effective Practice and Organization of Care (EPOC) group regarding randomized study designs (RCTs), controlled clinical trials (CCTs), controlled before and after studies (CBA), and interrupted time series (ITS) will be included [8]. In addition, we will also include cohort or panel studies, case-control studies, and relevant non-comparative publications such as case reports. A case report is a document that provides details about how a study was conducted and its subsequent findings and a panel study is a longitudinal study in which variables are measured on the same units over time.

We will categorize and analyze the review findings according to the study design employed, the study quality (low versus high quality studies) and the method reported in the primary studies. We will present the results of studies in tabular form.

### Types of institutions and participants

• All relevant hospital categories, hospital professionals and patients

• All categories of hospitals

• Individual hospital departments

• Hospital employees, such as health professionals, administrative staff and support staff

• Patients

• Management and stakeholders

### Types of methods reported

Following our broad review questions, we will include all relevant and objectively stated methods for evaluating hospital mergers and hospital cooperation strategies as well as objectively reported methods of hospital cooperation. Cooperation activities include the concepts of Hospital Cooperation; Health Facility and Hospital Merger; Hospital Shared Services; Health Care Coalitions; Health Facility Moving [6] and Hospital Fusion [7]. These activities also include hospital alliances, partnerships and networks.

### Types of outcome measures

All objectively measured outcomes of hospital mergers and other cooperation strategies (see background, definitions and review question) will be included and presented in tabular form. We will include patient outcomes, professional practice, including economic measures (that is, quality of care, hospital costs, charges, and so on)

### Search methods for the identification of studies

We will search electronic databases using a strategy incorporating the broad definition and concepts with selected medical subject headings (MeSH) and free text. (Please see additional file 1, search concepts and additional file 2, PubMed search strategy).

The Database of Abstracts of Reviews of Effectiveness (DARE) will be searched for related reviews. The following electronic databases will be searched for primary studies:

• The EPOC Register

• The Cochrane Central Register of Controlled Trails

• Business Source Premier (via EBSCO)

• Centre for Reviews and Dissemination Databases (including NHS EED and HTA-Databases)

• PubMed (via NLM)

• Global Health Database

• Web of Science

Other search methods:

• Hand-searching of those documents that have not been indexed and/or published electronically (that is, grey literature)

• Searching the references cited of all papers, relevant reviews or guidelines identified

• Contacting authors of relevant papers for further information/further unpublished work

• Contacting authors of other reviews for further information

Additionally, we will contact professional organizations and associations regarding relevant evaluation and cooperation methods in which they were involved.

We will search electronic databases using a strategy incorporating the methodological components of the review question combined with selected MesH terms and free text terms relating to hospital cooperation activities (see background, definitions and additional file 1, search concepts). This search strategy will be translated into the other databases using the appropriate controlled vocabulary as applicable. We will not use language restrictions.

For the PubMed (via NLM) search strategy, please see additional file 2.

## Methods of the review

### Screening

All titles and abstracts will be included in a reference management database; duplicates will be deleted. Two review authors will independently screen all titles and abstracts (TR and UR) to assess which studies meet the inclusion criteria. We will retrieve the full text copies of all potentially relevant papers. Disagreement on inclusion will be resolved by a third member of the research team (HB).

### Data management

We will record and report details on the number of retrieved references, the number of full text papers obtained and the number of included and excluded articles. We will manage this data in EndNote and RevMan and the criteria for excluding retrieved studies in phase 2 will be stated.

### Data extraction

Data will be extracted using a standardized data extraction sheet and directly apply it to study reports. When necessary, we will seek additional information from the authors of the primary studies. Relevant data will be entered into the RevMan software.

### Risk of bias assessment

Two independent review authors will assess the methodological quality of all included studies, using the EPOC checklist for the assessment of methodological quality of studies [8]. The methodological quality of included studies will be assessed and we will categorize them into three classes: A (low risk of bias), B (moderate risk of bias) and C (high risk of bias). We will refer unresolved disagreement on risk of bias to a third review author. We will exclude studies classified as high risk of bias.

### Data analysis

For the first two review questions, all relevant data will be extracted and presented in tabular form. Relevant findings will be categorized and synthesized in the form of a narrative summary using text and evidence tables according to the method reported in the primary study [9].

Regarding the third review question that is the effects of hospital cooperation, data in natural units will be reported. In the case of missing standard deviation, the appropriate transformation will be undertaken [10]. If possible, a summarized effect size, that is a weighted mean difference with 95% confidence intervals, will be estimated for continuous outcome measures and a weighted odds ratio as a risk ratio for dichotomous outcomes [11]. Economic measures will be assessed and calculated in the individual studies. Financial data will be presented in US$ for a common price year and will be adjusted for inflation by applying country-specific discount rates [12]. Additionally, we will provide the undiscounted cost data to allow readers to recalculate the results using any discount rate. Studies reporting in other currencies will be converted to US$ [13].

### Combining studies

We will make an assessment of the reported method and effects, based upon the quality, size, and direction of effects observed. Studies will be grouped following the method reported in the primary study. For the first two review questions, results will be synthesized and each method of hospital cooperation will be reported in the form of a narrative summary using text and tables. The review findings will also be assessed concerning the transferability and practical relevance of the published methods.

For the third review question, the results of studies will be depicted in tabular form. We expect to find both statistical and contextual heterogeneity, given the range of outcomes measured and the many different settings and types of professionals and patients included. This makes it improbable that statistical pooling will be feasible, but if there appears to be a body of studies amenable to meta-analysis, then their results will be displayed graphically and viewed to assess heterogeneity.

### Ongoing studies

We will describe identified ongoing studies, where available, detailing the primary author, research question(s), methods and outcome measures together with an estimate of the reporting date.

## Discussion

Overall, the systematic review aims to identify, assess and synthesize the evidence to underpin hospital cooperation activities as defined in this protocol. As a result, the review will provide an evidence-base for partnerships, alliances or other fields of cooperation in a hospital setting, developed on the basis of the review findings and conclusions.

## Competing interests

The authors declare that they have no competing interests.

## Authors' contributions

All review authors have contributed to the production of the protocol and all authors read and approved the final manuscript. TR led the writing of the protocol, all other review authors provided comment and feedback. For the full review: TE and BR have developed and will run the search strategy. TR and UR will screen all titles and abstracts for eligibility. TR, UR and BR will assess all primary studies for eligibility in review phase II. All review authors will abstract data, undertake analysis and write up the review. DP will take the leadership regarding additional search strategies as defined in this review protocol. GB will give advice on the methodological issues and will take the lead on the statistical analysis. HB will act as arbitrator should disagreement arise and will give advice on methodological issues. PD and GP will assess all full text studies in the second review stage about the practical relevance of the published methods. TR will lead the writing of the full review. PD and GP will critically appraise the review findings and conclusions, that is, to assess the transferability of the international evidence.

## Supplementary Material

Additional file 1 **Electronic search concepts**. The depicted search concepts were used to develop the search strategy. Click here for file

Additional file 2 **PubMed search strategy**. The depicted search strategy will be used to search PubMed. Click here for file
