# Supplementary figures and images for: Methods for the evaluation of hospital cooperation activities (Systematic review protocol)
Source: Syst Rev. 2012 Feb 10;1:11. doi: 10.1186/2046-4053-1-11 (PMC3351703; doi:10.1186/2046-4053-1-11)

## ECOMA – PubMed Search Strategy

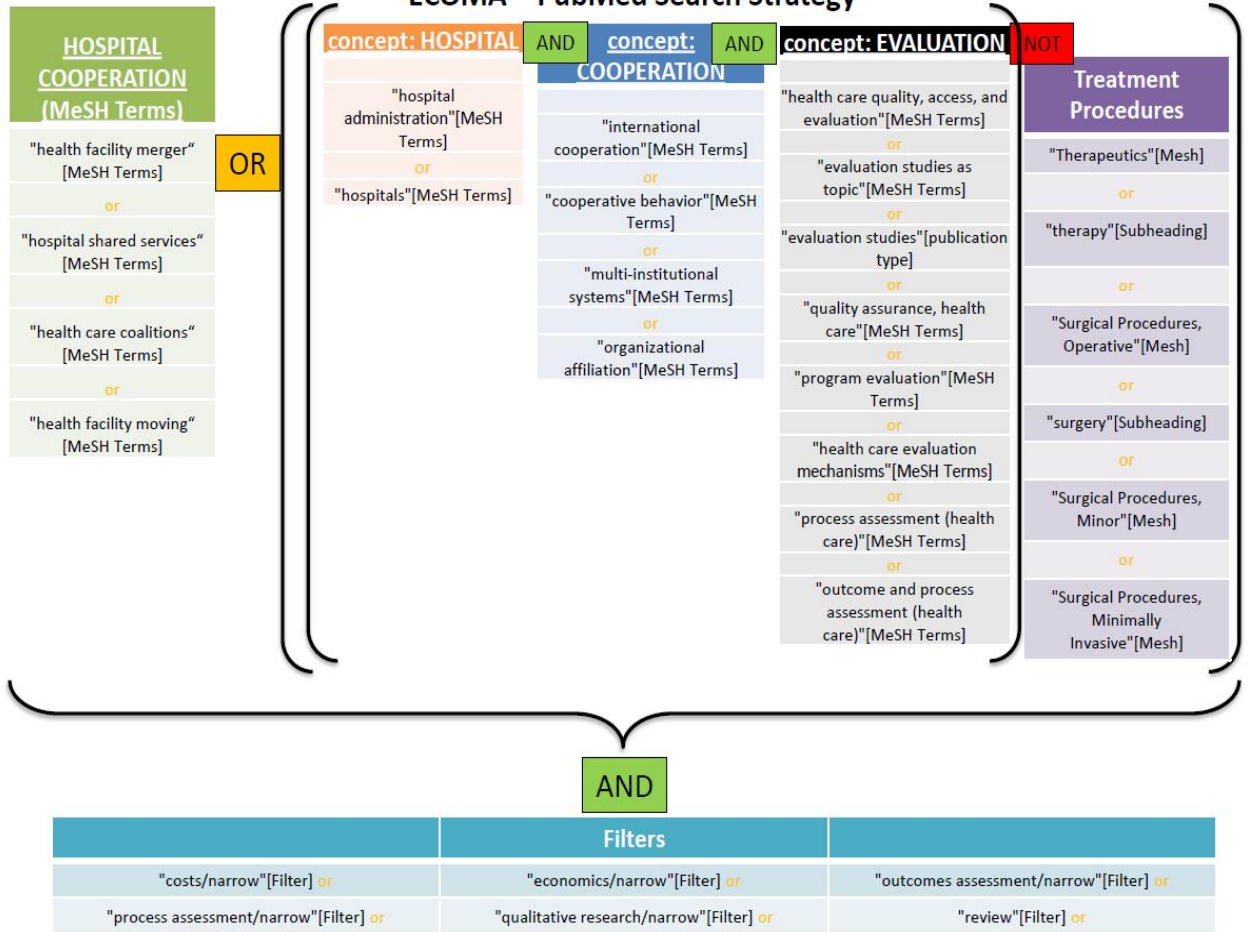

Supplement: Additional file 1 — Electronic search concepts. The depicted search concepts were used to develop the search strategy. [file 2046-4053-1-11-S1.PDF]
