# Supplementary material for: Methods for the evaluation of hospital cooperation activities (Systematic review protocol)
Source: Syst Rev. 2012 Feb 10;1:11. doi: 10.1186/2046-4053-1-11 (PMC3351703; doi:10.1186/2046-4053-1-11)
Supplement: Additional file 2 — PubMed search strategy. The depicted search strategy will be used to search PubMed. [file 2046-4053-1-11-S2.PDF]

We will search PubMed (via NLM) using the following search strategy:

Database: PubMed via National Library of Medicine (NLM) 1950 – week 12 2011 (last update)

1) "Health Facility Merger"[Mesh]
2) "Hospital Shared Services"[Mesh]
3) "Health Care Coalitions"[Mesh]
4) "Health Facility Moving"[Mesh]
5) "Hospital Administration"[Mesh]
6) "Hospitals"[Mesh]
7) "International Cooperation"[Mesh]
8) "Cooperative Behavior"[Mesh]
9) "Multi-Institutional Systems"[Mesh]
10) "Organizational Affiliation"[Mesh]
11) "Health Care Quality, Access, and Evaluation"[Mesh]
12) "Evaluation Studies as Topic"[Mesh]
13) "Evaluation Studies"[Publication Type]
14) "Quality Assurance, Health Care"[Mesh]
15) "Program Evaluation"[Mesh]
16) "Health Care Evaluation Mechanisms"[Mesh]
17) "Process Assessment (Health Care)"[Mesh]
18) "Outcome and Process Assessment (Health Care)"[Mesh]
19) "Therapeutics"[Mesh]
20) "therapy"[Subheading]
21) "Surgical Procedures, Operative"[Mesh]
22) "surgery"[Subheading]
23) "Surgical Procedures, Minor"[Mesh]
24) "Surgical Procedures, Minimally Invasive"[Mesh]
25) "costs/narrow"[Filter]
26) "economics/narrow"[Filter]
27) "outcomes assessment/narrow"[Filter]
28) "process assessment/narrow"[Filter]
29) "qualitative research/narrow"[Filter]
30) "review"[Filter]
31) (((#1) OR #2) OR #3) OR #4
32) (#5) OR #6
33) (((#7) OR #8) OR #9) OR #10
34) (((((((#11) OR #12) OR #13) OR #14) OR #15) OR #16) OR #17) OR #18
35) (((((#19) OR #20) OR #21) OR #22) OR #23) OR #24
36) (((((#25) OR #26) OR #27) OR #28) OR #29) OR #30
37) ((#32) AND #33) AND #34
38) (#37) NOT #35
39) (#31) OR #38
40) (#39) AND #36
